# Ureteral Involvement and Diabetes Increase the Risk of Subsequent Bladder Recurrence after Nephroureterectomy for Upper Urinary Tract Urothelial Carcinoma

**DOI:** 10.1155/2015/527976

**Published:** 2015-03-31

**Authors:** Che-Yuan Hu, Yu-Chieh Tsai, Shuo-Meng Wang, Chao-Yuan Huang, Huai-Ching Tai, Chung-Hsin Chen, Yeong-Shiau Pu, Wei-Chou Lin, Kuo-How Huang

**Affiliations:** ^1^Department of Urology, National Taiwan University Hospital, Taipei 100, Taiwan; ^2^Department of Oncology, National Taiwan University Hospital, Taipei 100, Taiwan; ^3^Department of Pathology, National Taiwan University Hospital, Taipei 100, Taiwan

## Abstract

*Objectives.* To investigate the prognostic factors for bladder recurrence after radical nephroureterectomy (RNU) in patients with upper urinary tract urothelial carcinoma (UUT-UC). *Methods.* From 1994 to 2012, 695 patients with UUT-UC treated with RNU were enrolled in National Taiwan University Medical Center. Among them, 532 patients with no prior bladder UC history were recruited for analysis. We assessed the impact of potentially prognostic factors on bladder recurrence after RNU. *Results.* The median follow-up period was 47.8 months. In the Cox model, ureteral involvement and diabetes mellitus (DM) were significantly associated with a higher bladder recurrence rate in the multivariate analysis (hazard ratio [HR]: 1.838; *P* = 0.003 and HR: 1.821; *P* = 0.010, resp.). In the Kaplan-Meier analysis, DM patients with concomitant ureteral UC experienced about a threefold increased risk of bladder recurrence as compared to those without both factors (HR: 3.222; *P* < 0.001). Patients with either of the two risk factors experienced about a twofold increased risk as compared to those without both factors (with DM, HR: 2.184, *P* = 0.024; with ureteral involvement, HR: 2.006, *P* = 0.003). *Conclusions.* Ureteral involvement and DM are significantly related to bladder recurrence after RNU in patients with UUT-UC.

## 1. Introduction

Upper urinary tract urothelial tumors represent approximately 5% of all urothelial tumors in the world [1]. However, 20–30% of all urothelial tumors are located in the upper tract in Taiwan [2]. The use of aristolochic acid contaminated Chinese herbal medicine has been reported to be associated with the high incidence of upper urinary tract urothelial carcinoma (UUT-UC) in Taiwan [3]. Radical nephroureterectomy (RNU) and bladder cuff resection is currently the standard treatment for localized UUT-UC [4]. Bladder recurrence has been mentioned as one of the major concerns with regard to oncological outcomes after RNU [5]. Bladder recurrence undoubtedly increases the disease burden and worsens the prognosis of UUT-UC after RNU. Therefore, regular cystoscopy follow-up after RNU is recommended in all UUT-UC patients [4]. However, cystoscopy itself is an invasive and costly procedure. It is thus extremely important to investigate the risk factors and identify those patients at high risk of bladder recurrence following RNU so that physicians can provide a closer follow-up schedule and adjuvant intravesical therapy to reduce bladder recurrence [6].

Numerous studies have been conducted to elucidate the prognostic factors for bladder recurrence after RNU for UUT-UC, although the results remain controversial [7]. Additionally, various bladder recurrence rates have been reported, ranging from 20 to 50% after RNU for primary UUT-UC [7]. Many of the results from previous studies vary because of inconsistencies in patient selection with regard to follow-up period or exclusion of prior bladder UC history [7].

This study aimed to retrospectively investigate patients with UUT-UC treated by RNU and bladder cuff resection to determine the impact of potential risk factors on subsequent bladder recurrence after RNU.

## 2. Materials and Methods

From July 1994 to April 2012, a total of 695 patients with UUT-UC treated with RNU were enrolled in National Taiwan University Medical Center. Among them, 532 patients with no prior bladder UC history were recruited for final analysis. RNU was performed by open or laparoscopic method via transperitoneal or retroperitoneal route. Dissection of regional lymph nodes was performed in patients with suspicious lymphadenopathy on preoperative imaging studies or those suspected of having enlarged nodes during intraoperative inspection.

Clinical data were collected by a retrospective review of medical records. Follow-up included cystoscopy and urine cytology at three-month intervals for the first year, at six-month intervals for the subsequent two years, and then annually. Computed tomography and excretory urography were performed annually. Adjuvant systemic and intravesical chemotherapy were only administered in patients with the clinical evidence of recurrence. When bladder tumor was noted by cystoscopy or image follow-up, transurethral resection of the bladder tumor was performed. Recurrence was judged by histological proof. Pathological staging was performed according to the 2009 TNM staging system [4]. If carcinoma in situ existed concomitantly with Ta (noninvasive urothelial carcinoma), it would be classified as “Tis.” However, if carcinoma in situ existed concomitantly with T1~T4 (invasive urothelial carcinoma), it would be classified as T1 to T4 according to the most severe stage from all lesions of the specimen. Histological grading followed the 2004 World Health Organization grade classification [8].

The potentially prognostic factors assessed included age (older versus younger than 65 years), gender (male versus female), body mass index (BMI), pT stage (Ta-1, T2, T3-4, and Tis), grade (low versus high), tumor site (with versus without ureteral involvement), tumor multiplicity (yes versus no), residency in arsenic-endemic area (yes versus no), Chinese herbal medicine use (yes versus no), cigarette smoking (yes versus no), diabetes mellitus (DM) (yes versus no), hypertension (yes* versus* no), estimated glomerular filtration rate (eGFR) (<60 mL/min versus ≥60 mL/min), hemodialysis history before RNU (yes versus no), operation method, and pN stage (N0 versus N1–3). The formula used to calculate BMI was body weight (kg)/body height squared (m^2^). Hypertension was defined as systolic blood pressure (SBP) ≥ 140 mmHg, diastolic blood pressure (DBP) ≥ 90 mmHg, or a history of hypertension in the subject's medical records. DM was defined as 2 h-PG ≥ 11.1 mmol/L, FPG ≥ 7.0 mmol/L, or a history of type 2 diabetes mellitus in the subject's medical records [9]. Multiplicity was defined as the presence of more than one tumor with healthy mucosa between them (≥two tumor foci). The prognostic implications of these factors with regard to bladder recurrence-free survival were analyzed. We defined “recurrence-free survival time” as the period between the date of the RNU for primary UUT-UC and the date of the first bladder recurrence.

Statistical analyses were performed using SPSS software (SPSS Inc., Chicago, IL, USA). Continuous data were expressed as mean ± standard deviation (SD), and percentages were calculated for categorical variables. The Mann-Whitney *U* test was used to compare the mean of continuous data, whereas the chi-square test was used to analyze categorical proportions. Univariate analyses and bladder recurrence-free survival curves were generated using the Kaplan-Meier method. Multivariate analyses were performed using Cox's proportional hazards model to estimate the hazard ratio and 95% confidence interval. Two-tailed *P* < 0.05 was considered statistically significant.

## 3. Results

A total of 532 patients with UUT-UC formed the study sample. The mean age was 65.3 ± 11.7 years. The median follow-up period was 47.8 months (range: 3–210). Bladder recurrence developed in 156 (29.3%) patients.  Table 1 outlines the patients' clinicopathological characteristics stratified by the presence of ureteral involvement. There were significant differences in tumor multiplicity (*P* < 0.001), residence in arsenic-endemic area (*P* = 0.035), grading (*P* = 0.004), and pT stage (*P* < 0.001) between the two groups, ureteral involvement versus no involvement. The mean time to bladder recurrence was 18.8 ± 27.0 months in the patients with ureteral involvement and 19.5 ± 24.4 months in those without it.

**Figure 1:**
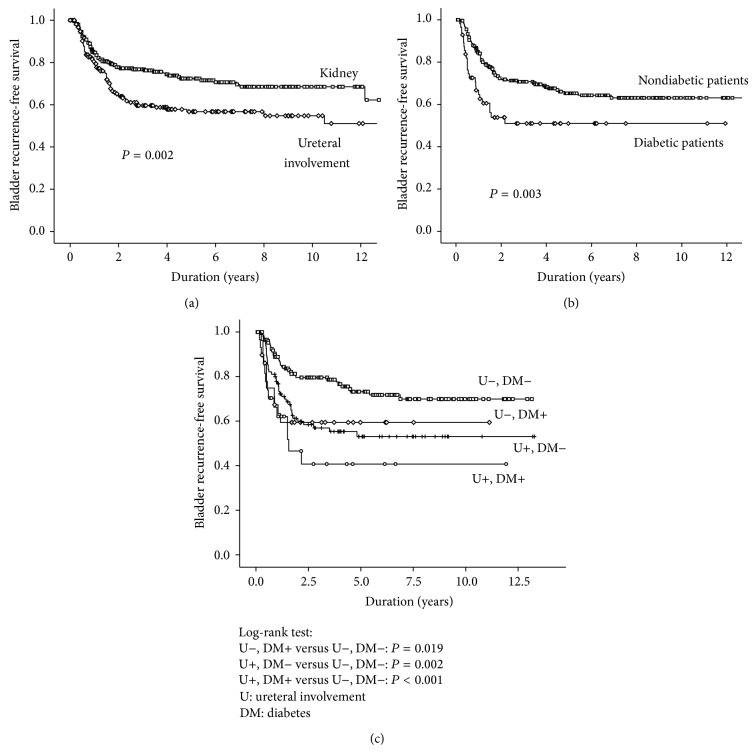
Kaplan-Meier analysis on bladder recurrence-free survival, (a) kidney versus ureteral involvement, (b) diabetes mellitus (DM) versus non-DM, (c) stratification by the presence of ureteral involvement or DM.

Table 2 shows the univariate and multivariate analyses for variables associated with subsequent bladder recurrence after RNU. The multivariate analyses revealed DM (HR: 1.821; *P* = 0.010) and ureteral involvement (HR: 1.838; *P* = 0.003) were significant risk factors associated with bladder recurrence. The Kaplan-Meier survival curve and log-rank test on bladder recurrence-free survival in UUT-UC patients stratified by the presence of ureteral involvement and DM are shown in Figures 1(a) and 1(b), respectively. UUT-UC patients with ureteral involvement had a higher risk of bladder recurrence after RNU compared to those without it (*P* = 0.002). The one-, two-, and five-year bladder recurrence-free survival rates were 81%, 65%, and 57% in patients with ureteral involvement and 85%, 78%, and 72% in those without ureteral involvement, respectively. In addition, there were significant differences in bladder recurrence after RNU for UUT-UC between DM patients and non-DM subjects (*P* = 0.003).

We further analyzed the bladder recurrence-free survival in UUT-UC patients by substratification of the presence of ureteral involvement and DM. The results are shown in Figure 1(c). The bladder recurrence-free survival was the worst in patients with concomitant diabetes and ureteral involvement, followed by those with one of the two risk factors. The prognosis was the best in those without any of the two risk factors. Patients with concomitant diabetes and ureteral UC had a significantly lower bladder recurrence-free survival rate as compared to those without any of the two risk factors (*P* < 0.001). Similarly, patients with either of the two risk factors witnessed a significantly increased risk of bladder recurrence as compared to those without either of the two risk factors (with DM, *P* = 0.019; with ureteral involvement, *P* = 0.002, resp.). The five-year recurrence-free survival rate for patients with concomitant ureteral involvement and DM was 41% compared with 73% for patients without any of the two risk factors.  Table 3 shows that patients with concomitant ureteral involvement and DM experienced about a threefold increased risk of developing bladder recurrence compared to those with neither of the two risk factors (HR: 3.222; *P* < 0.001). Consistently, patients with either DM or ureteral involvement experienced an approximately twofold increased risk of developing bladder recurrence compared to those with neither of the risk factors (with DM, HR: 2.184, *P* = 0.024; with ureteral involvement, HR: 2.006, *P* = 0.003, resp.).

## 4. Discussion

The present study identified two significant factors associated with bladder recurrence of UUT-UC after RNU: DM and ureteral involvement. The overall bladder recurrence rate in our study with long-term follow-up was 29.3%, consistent with the results of previous reports, which ranged from 20 to 50% [7]. In agreement with most previous studies, we also found that most of the bladder recurrence occurred within the first two years after RNU [10]. The National Comprehensive Cancer Network (NCCN) guidelines published in 2012 recommended cystoscopy and urinary cytology checkup every three months for one year and then at increasing intervals after NU for primary UUT-UC [11]. Our findings support the follow-up protocol recommended by NCCN. However, in UUT-UC patients with concomitant DM and ureteral involvement, a closer follow-up schedule and even adjuvant intravesical therapy may need to be considered, as they are at higher risk of bladder recurrence.

A history of bladder UC has been reported to be a significant risk factor for bladder recurrence after UUT-UC treatment in several previous studies [12]. We thus excluded patients with a prior history of bladder UC to minimize the bias from study subjects with primary UUT-UC. Moreover, our study subjects were representative of a special population in Taiwan, one which comes from an area with a high prevalence of UUT-UC. The etiologies responsible for the high prevalence of UUT-UC have been described as arsenic contaminated drinking water and the use of aristolochic-containing Chinese herbal medicine. Based on this, we included all potentially relevant risk factors in Taiwan, such as smoking, residency in an arsenic-endemic area, and use of Chinese herbal medicine, in order to determine all possible risk factors [13–15].

The association of DM and cancer occurrence has been described in various sites, such as liver, pancreas, colon, esophagus, kidney, blood, breast, endometrium, and bladder [16]. DM is considered to be a low-grade inflammation that is associated with reduced antioxidant capacity and an adverse secretory profile of cytokines [17]. Oxidative stress can result in genomic instability and DNA mutations, and cytokines such as interleukin 6, tumor necrosis factor-alpha, and plasminogen activator inhibitor-1 are thought to be involved in malignant transformation [18–20]. In addition, DM patients often tend to suffer from bacterial cystitis, and asymptomatic bacteriuria and symptomatic urinary tract infection are more common in DM women compared to healthy women [21]. Damaged urothelium and glycosaminoglycan and mucopolysaccharide layers on the surface of the urinary bladder may enhance the risk of UC cell adherence and implantation [22]. Both epidemiological [23] and animal studies [24] reveal that urinary tract infection has a significant impact on the subsequent occurrence of bladder cancer. On the other hand, the relationship between the use of insulin sensitizing peroxisome proliferator-activated receptor gamma agonists for the treatment of type II DM, such as pioglitazone, and the risk of bladder cancer is still under debate [25]. A study using the National Health Insurance database in Taiwan revealed that, among 422 patients using pioglitazone, none had developed bladder cancer during the three-year observation period [26]. However, the current study had no data on its diabetic subjects' history of pioglitazone usage. Although DM is actually an independent predictor for bladder UC recurrence after RNU, the role that pioglitazone plays in the linkage between diabetes and bladder recurrence in our cohort thus remains unclear.

A number of studies have described the location of the primary tumor as a risk factor for oncological outcomes and subsequent bladder recurrence for UUT-UC. While some research has indicated that synchronous ureter and renal pelvic UC is a significant risk factor [27–30], others have found that tumor multiplicity is being a more important predictor than tumor locations [31, 32]. These inconsistent results may be due to variations in the definitions of multiplicity and location in the related literature. The current study showed that ureteral involvement increased the risk of bladder recurrence by 1.8-fold (HR: 1.838; *P* = 0.003). In contrast, multiplicity did not emerge as a significant predictor of bladder recurrence.

Two hypotheses have been proposed to explain subsequent bladder recurrence after RNU: intraluminal seeding and implantation of cancer cells and field cancerization [33]. Some molecular studies on UUT-UC and subsequent bladder recurrence suggest that seeding and intraepithelial spread are major mechanisms for the multifocal development of UC [34, 35]. We hypothesized that the underlying disease of DM creates a damaged mucosal layer over the bladder wall and that urothelial carcinoma located in the lower ureter facilitates tumor cell seeding to the bladder due to the adjacent location. These two factors thus increased the possibility of bladder recurrence.

A history of hemodialysis was found to have a borderline significant association with bladder recurrence in our cohort (multivariate analysis, HR: 1.791, *P* = 0.055). Previous studies have documented that end-stage renal disease (ESRD) patients on renal replacement therapy were at increased risk of urothelial carcinoma [36]. Residency in an arsenic-endemic area was not associated with the risk of bladder recurrence after RNU in the current study. An earlier study [2] had the same result, and this might be explained by the use of a tap water supply system (arsenic concentration 10 *μ*g/L) instead of artesian-well water (arsenic concentration 700 to 930 *μ*g/L) in our arsenic-endemic area since the early 1970s [37]. In contrast to previous reports, concomitant CIS was not significantly associated with bladder recurrence after RNU in our study. This could be due to the low incidence of CIS in our dataset compared to that in prior studies, indicating that the CIS prevalence rate was approximately 30% in UUT-UC [38].

This study has several limitations that should be taken into consideration. First, it was a retrospective study in a single center setting and some data was missing for some of the variables. We also lacked some detailed clinical information, such as diabetes duration and control, smoking status, being a current or former smoker, tumor size, the duration of residence in an arsenic-endemic area, and the methods of distal ureteral management used. In addition, pathological diagnosis was not performed by a central pathologist. However, this study utilized a large patient number, longer follow-up period, and homogeneous patient sample and thus is still able to provide important information which may be beneficial to clinicians and serve as a reference in relation to further studies.

## 5. Conclusions

We identified ureteral involvement and DM as risk factors of bladder recurrence after RNU. Based on these findings, physicians can provide more individualized follow-up protocols and identify those patients who may benefit from prophylactic intravesical therapy according to the risk stratification.

## Figures and Tables

**Table 1:** Clinicopathological characteristics stratified by ureteral involvement in 532 patients with UUT-UC treated by RNU.

Variable	No ureteral involvement, *n* (%)	Ureteral involvement, *n* (%)	*P*
	299 (56.2)	233 (43.8)	
Median follow-up period (months)	51.5	43.9	0.227
Age (years)			0.685
<65	131 (43.8)	98 (42.1)	
≥65	168 (56.2)	135 (57.9)	
BMI (mean ± SD)	24.3 ± 4.2	23.5 ± 4.3	0.185
Gender			0.533
Male	157 (52.5)	116 (49.8)	
Female	142 (47.5)	117 (50.2)	
Residency			0.035^*^
Nonarsenic	295 (98.7)	223 (95.7)
Arsenic	4 (1.3)	10 (4.3)
Smoking			0.801
No	232 (78.1)	176 (77.2)	
Yes	65 (21.9)	52 (22.8)	
Herb			0.058
No	174 (93.0)	118 (86.8)
Yes	13 (7.0)	18 (13.2)
DM			0.132
No	158 (84.5)	106 (77.9)	
Yes	29 (15.5)	30 (22.1)	
Hypertension			0.363
No	122 (65.2)	82 (60.3)	
Yes	65 (34.8)	54 (39.7)	
eGFR (mL/min)			0.105
<60	51 (33.3)	30 (24.4)	
>60	102 (66.7)	93 (75.6)	
Hemodialysis			0.065
No	277 (92.6)	204 (87.9)	
Yes	22 (7.4)	28 (12.1)	
Operation method			0.236
Open			
Transperitoneal	27 (23.5)	14 (19.4)	
Retroperitoneal	27 (23.5)	27 (37.5)	
Laparoscopy			
Transperitoneal	35 (30.4)	18 (25.0)	
Retroperitoneal	26 (22.6)	13 (18.1)	
Multiplicity			<0.001^*^
No	164 (87.7)	91 (66.9)	
Yes	23 (12.3)	45 (33.1)	
Grade			0.004^*^
Low	150 (54.9)	86 (41.7)	
High	123 (45.1)	120 (58.3)	
T stage			<0.001^*^
Ta~T1	146 (56.4)	87 (39.5)	
T2	21 (8.1)	62 (28.2)	
T3~4	89 (34.4)	67 (30.5)	
Tis	3 (1.2)	4 (1.8)	
N stage			0.278
N0	218 (90.5)	182 (93.3)	
N1, N2, N3	23 (9.5)	13 (6.7)	

Data expressed as mean ± SD or numbers (%), BMI: body mass index, DM: diabetes mellitus, eGFR: estimated glomerular filtration rate, nonarsenic: non-black-foot disease endemic area, and arsenic: black foot disease endemic area.

^*^Significant difference, *P* < 0.05.

**Table 2:** Univariate and multivariate analyses for variables significantly associated with subsequent bladder recurrence after RNU in 532 patients with UUT-UC.

Variable	Univariate		Multivariate	
	HR (95% CI)	*P*	HR (95% CI)	*P*
Age (years)				
<65	1	—		
≥65	0.819 (0.594–1.128)	0.222		
Gender				
Female	1	—		
Male	1.210 (0.878–1.669)	0.245		
Smoking				
No	1	—		
Yes	1.221 (0.838–1.778)	0.298		
BMI	0.967 (0.909–1.028)	0.277		
eGFR (mL/min)				
<60	1	—		
>60	1.347 (0.757–2.396)	0.312		
Hemodialysis				
No	1	—	1	—
Yes	1.603 (0.990–2.595)	0.055	1.791 (0.988–3.244)	0.055
Chinese herbal medicine use				
No	1	—	1	—
Yes	1.851 (1.012–3.386)	0.046^*^	1.387 (0.731–2.632)	0.316
Hypertension				
No	1	—		
Yes	1.292 (0.868–1.922)	0.207		
DM				
No	1	—	1	—
Yes	1.953 (1.241–3.074)	0.004^*^	1.821 (1.152–2.878)	0.010^*^
Residency				
Nonarsenic	1	—		
Arsenic	0.775 (0.247–2.434)	0.662		
OP method				
Transperitoneal	1	—		
Retroperitoneal	1.097 (0.674–1.786)	0.708		
OP method				
Open	1	—		
Laparoscopy	1.420 (0.868–2.323)	0.162		
Grade				
Low	1	—		
High	0.994 (0.705–1.402)	0.974		
Ureteral involvement				
No	1	—	1	—
Yes	1.639 (1.189–2.260)	0.003^*^	1.838 (1.233–2.741)	0.003^*^
Multiplicity				
No	1	—		
Yes	1.499 (0.953–2.356)	0.080		
T stage				
Ta, 1	1	—		
T2	1.135 (0.727–1.771)	0.578		
T3, 4	0.914 (0.610–1.368)	0.661		
Tis	2.110 (0.770–5.785)	0.147		
N stage				
N0	1	—		
N1, 2, 3	0.394 (0.125–1.239)	0.111		

BMI: body mass index, DM: diabetes mellitus, HR: Hazard ratio, and CI: confidence interval.

^*^Significant difference, *P* < 0.05.

**Table 3:** The hazard ratio based on the presence of ureteral involvement and diabetes in the Kaplan-Meier analysis.

Ureteral involvement	DM	*P *	HR	95% CI	
No	No		1		
No	Yes	0.024^*^	2.184	1.110	4.295
Yes	No	0.003^*^	2.006	1.277	3.149
Yes	Yes	<0.001^*^	3.222	1.732	5.991

DM: diabetes mellitus, HR: Hazard ratio, and CI: confidence interval.

^*^Significant difference, *P* < 0.05.

## References

[1] Jemal A., Tiwari R. C., Murray T. (2004). Cancer statistics, 2004. *CA—Cancer Journal for Clinicians*.

[2] Yang M.-H., Chen K.-K., Yen C.-C. (2002). Unusually high incidence of upper urinary tract urothelial carcinoma in Taiwan. *Urology*.

[3] Chen C.-H., Dickman K. G., Huang C.-Y. (2013). Aristolochic acid-induced upper tract urothelial carcinoma in Taiwan: clinical characteristics and outcomes. *International Journal of Cancer*.

[4] Rouprêt M., Zigeuner R., Palou J. (2011). European guidelines for the diagnosis and management of upper urinary tract urothelial cell carcinomas: 2011 update. *European Urology*.

[5] Kamihira O., Hattori R., Yamaguchi A. (2009). Laparoscopic radical nephroureterectomy: a multicenter analysis in Japan. *European Urology*.

[6] O'Brien T., Ray E., Singh R., Coker B., Beard R. (2011). Prevention of bladder tumours after nephroureterectomy for primary upper urinary tract urothelial carcinoma: a prospective, multicentre, randomised clinical trial of a single postoperative intravesical dose of mitomycin C (the ODMIT-C Trial). *European Urology*.

[7] Kauffman E. C., Raman J. D. (2008). Bladder cancer following upper tract urothelial carcinoma. *Expert Review of Anticancer Therapy*.

[8] MacLennan G. T., Kirkali Z., Cheng L. (2007). Histologic grading of noninvasive papillary urothelial neoplasms. *European Urology*.

[9] Yang G.-H., Wu J.-S., Yang Y.-C., Huang Y.-H., Lu F.-H., Chang C.-J. (2014). Gastric *Helicobacter pylori* infection associated with risk of diabetes mellitus, but not prediabetes. *Journal of Gastroenterology and Hepatology*.

[10] Kang C.-H., Yu T.-J., Hsieh H.-H. (2003). The development of bladder tumors and contralateral upper urinary tract tumors after primary transitional cell carcinoma of the upper urinary tract. *Cancer*.

[11] Clark P. E., Agarwal N., Biagioli M. C. (2013). Bladder cancer. *Journal of the National Comprehensive Cancer Network*.

[12] Xylinas E., Colin P., Audenet F. (2013). Intravesical recurrence after radical nephroureterectomy for upper tract urothelial carcinomas: predictors and impact on subsequent oncological outcomes from a national multicenter study. *World Journal of Urology*.

[13] Rink M., Xylinas E., Margulis V. (2013). Impact of smoking on oncologic outcomes of upper tract urothelial carcinoma after radical nephroureterectomy. *European Urology*.

[14] Tan L.-B., Chen K.-T., Guo H.-R. (2008). Clinical and epidemiological features of patients with genitourinary tract tumour in a blackfoot disease endemic area of Taiwan. *BJU International*.

[15] Chen C.-H., Dickman K. G., Moriya M. (2012). Aristolochic acid-associated urothelial cancer in Taiwan. *Proceedings of the National Academy of Sciences of the United States of America*.

[16] Habib S. L., Rojna M. (2013). Diabetes and risk of cancer. *ISRN Oncology*.

[17] Sakkinen P. A., Wahl P., Cushman M., Lewis M. R., Tracy R. P. (2000). Clustering of procoagulation, inflammation, and fibrinolysis variables with metabolic factors in insulin resistance syndrome. *American Journal of Epidemiology*.

[18] Iliopoulos D., Hirsch H. A., Struhl K. (2009). An epigenetic switch involving NF-*κ*B, Lin28, Let-7 MicroRNA, and IL6 links inflammation to cell transformation. *Cell*.

[19] Balkwill F. (2006). TNF-*α* in promotion and progression of cancer. *Cancer and Metastasis Reviews*.

[20] Ulisse S., Baldini E., Sorrenti S., D'Armiento M. (2009). The urokinase plasminogen activator system: a target for anti-cancer therapy. *Current Cancer Drug Targets*.

[21] Geerlings S. E., Stolk R. P., Camps M. J. L., Netten P. M., Collet T. J., Hoepelman A. I. M. (2000). Risk factors for symptomatic urinary tract infection in women with diabetes. *Diabetes Care*.

[22] See W. A., Chapman P. H. (1987). Heparin prevention of tumor cell adherence and implantation on injured urothelial surfaces. *Journal of Urology*.

[23] Kantor A. F., Hartge P., Hoover R. N., Narayana A. S., Sullivan J. W., Fraumeni J. F. (1984). Urinary tract infection and risk of bladder cancer. *American Journal of Epidemiology*.

[24] Johansson S. L., Anderstrom C., Von Schultz L., Larsson P. (1987). Enhancement of N-[4-(5-nitro-2-furyl)-2-thiazolyl]formamide-induced carcinogenesis by urinary tract infection in rats. *Cancer Research*.

[25] Levin D., Bell S., Sund R. (2015). Pioglitazone and bladder cancer risk: a multipopulation pooled, cumulative exposure analysis. *Diabetologia*.

[26] Tseng C.-H. (2011). Diabetes and risk of bladder cancer: a study using the National Health Insurance database in Taiwan. *Diabetologia*.

[27] Fradet V., Mauermann J., Kassouf W. (2014). Risk factors for bladder cancer recurrence after nephroureterectomy for upper tract urothelial tumors: results from the Canadian Upper Tract Collaboration. *Urologic Oncology*.

[28] Xylinas E., Kluth L., Passoni N. (2014). Prediction of intravesical recurrence after radical nephroureterectomy: development of a clinical decision-making tool. *European Urology*.

[29] Terakawa T., Miyake H., Muramaki M., Takenaka A., Hara I., Fujisawa M. (2008). Risk factors for intravesical recurrence after surgical management of transitional cell carcinoma of the upper urinary tract. *Urology*.

[30] Hisataki T., Miyao N., Masumori N. (2000). Risk factors for the development of bladder cancer after upper tract urothelial cancer. *Urology*.

[31] Matsui Y., Utsunomiya N., Ichioka K. (2005). Risk factors for subsequent development of bladder cancer after primary transitional cell carcinoma of the upper urinary tract. *Urology*.

[32] Milojevic B., Djokic M., Sipetic-Grujicic S. (2011). Bladder cancer after managing upper urinary tract transitional cell carcinoma: risk factors and survival. *International Urology and Nephrology*.

[33] Harris A. L., Neal D. E. (1992). Bladder cancer—field versus clonal origin. *The New England Journal of Medicine*.

[34] Takahashi T., Kakehi Y., Mitsumori K. (2001). Distinct microsatellite alterations in upper urinary tract tumors and subsequent bladder tumors. *Journal of Urology*.

[35] Maomi L., Cannizzaro L. A. (1999). Identical clonal origin of synchronous and metachronous low-grade, noninvasive papillary transitional cell carcinomas of the urinary tract. *Human Pathology*.

[36] Lin H.-F., Li Y.-H., Wang C.-H., Chou C.-L., Kuo D.-J., Fang T.-C. (2012). Increased risk of cancer in chronic dialysis patients: a population-based cohort study in Taiwan. *Nephrology Dialysis Transplantation*.

[37] Wang S.-L., Li W.-F., Chen C.-J. (2011). Hypertension incidence after tap-water implementation: a 13-year follow-up study in the arseniasis-endemic area of southwestern Taiwan. *Science of the Total Environment*.

[38] Pieras E., Frontera G., Ruiz X., Vicens A., Ozonas M., Pizá P. (2010). Concomitant carcinoma in situ and tumour size are prognostic factors for bladder recurrence after nephroureterectomy for upper tract transitional cell carcinoma. *BJU International*.

